# Co-immunoprecipitation for identifying protein–protein interaction on lipid droplets

**DOI:** 10.52601/bpr.2024.240007

**Published:** 2024-04-30

**Authors:** Xiaochuan Fu, Shuyan Zhang, Pingsheng Liu

**Affiliations:** 1 Institute of Biophysics, Chinese Academy of Sciences, Beijing 100101, China; 2 University of Chinese Academy of Sciences, Beijing 100049, China; 3 Institute of Infectious Diseases, Beijing Key Laboratory of Emerging Infectious Diseases, Beijing Ditan Hospital, Capital Medical University, Beijing 100015, China; 4 Beijing Institute of Infectious Diseases, Beijing 100015, China

**Keywords:** Lipid droplet, Co-immunoprecipitation, Protein interaction

## Abstract

The lipid droplet (LD) is a conserved organelle that exists in almost all organisms, ranging from bacteria to mammals. Dysfunctions in LDs are linked to a range of human metabolic syndromes. The formation of protein complexes on LDs is crucial for maintaining their function. Investigating how proteins interact on LDs is essential for understanding the role of LDs. We have developed an effective method to uncover protein–protein interactions and protein complexes specifically on LDs. In this method, we conduct co-immunoprecipitation (co-IP) experiments using LD proteins extracted directly from isolated LDs, rather than utilizing proteins from cell lysates. To elaborate, we begin by purifying LDs with high-quality and extracting LD-associated proteins. Subsequently, the co-IP experiment is performed on these LD-associated proteins directly, which would enhance the co-IP experiment specificity of LD-associated proteins. This method enables researchers to directly unveil protein complexes on LDs and gain deeper insights into the functional roles of proteins associated with LDs.

## INTRODUCTION

The lipid droplet (LD) is a cellular organelle for lipid storage that is composed of a neutral lipid core, surrounded by a phospholipid monolayer membrane (Farese and Walther 2009; Fujimoto *et al.* 2008; Martin and Parton 2006; Yang *et al.* 2012). The existence of LDs was initially observed in 1674 by van Leeuwenhoek (Kernohan and Lepherd 1969; Xu *et al.* 2018). However, due to technological limitations, LDs were primarily considered passive lipid reservoirs and were overlooked by scientists for a long time. With advancements in LD isolation methods and MS technology, the proteomes of LDs were successfully identified around the year 2000 (Athenstaedt *et al.* 1999; Fujimoto *et al.* 2004; Liu *et al.* 2004). Over the past two decades, our laboratory and other groups have purified LDs from various species and studied their proteomes (Beller *et al.* 2006; Ding *et al.* 2012; Kalscheuer *et al.* 2001; Liu *et al.* 2004; Wan *et al.* 2007; Zhang *et al.* 2011, 2012). LDs have been increasingly recognized as key players in lipid metabolism, membrane trafficking, membrane biosynthesis, energy homeostasis and signal transduction (Bartz *et al.* 2007; Goodman 2008; Murphy *et al.* 2009; Ohsaki *et al.* 2009; Olzmann and Carvalho 2019; Zehmer *et al.* 2009).

LD-associated proteins are the executors of LD functions. Therefore, LD-associated proteins have been revealed as important regulators of lipid homeostasis. Specifically, the resident proteins on LDs, such as perilipin family proteins (PLINs) (Brasaemle *et al.* 1997; Greenberg *et al.* 1991, 1993; Jiang and Serrero 1992; Miura *et al.* 2002; Sztalryd *et al.* 2003; Wang *et al.* 2011), play critical roles in maintaining lipid homeostasis and governing LD functions within mammalian cells. Furthermore, enzymes involved in lipid metabolism, including ATGL, ACSLs, and HSL, actively participate in the mobilization of lipids (Egan *et al.* 1992; Fujimoto *et al.* 2007; Zimmermann *et al.* 2004). Moreover, certain LD proteins have been implicated in various metabolic syndromes. For instance, a genetic variation in PNPLA3 (I148M) is strongly linked to heightened hepatic fat accumulation (Romeo *et al.* 2008). Research by Wang *et al*. shows that this mutation regulates lipolysis by influencing the interaction between PNPLA3 and comparative gene identification-58 (CGI-58) (Wang *et al.* 2019). Overexpression of LD protein HSD17B13 has been associated with hepatic steatosis (Abul-Husn *et al.* 2018; Su *et al.* 2014). Consequently, it is imperative to acquire a comprehensive understanding of the regulatory mechanisms governing lipid homeostasis and the dynamics of LD-associated proteins.

Proteins often exert their functions by interacting with other proteins or forming complexes with various biological molecules. Accumulating evidence underscores that many lipid droplet proteins govern lipid homeostasis through interactions with other proteins or by forming complexes on LD. For instance, a genetic variation in PNPLA3 (I148M) disrupts ATGL activity by interacting with its cofactor, CGI-58 (Wang *et al.* 2019). LD-associated Rab18 binds to PLIN2 and ACSL3 to mediate lipid droplet dynamics (Deng *et al.* 2021). Therefore, it is essential to gain a comprehensive understanding of protein interactions/complexes on LDs to fully comprehend LD regulation.

Numerous methods are available for studying protein interactions, including yeast two-hybrid (Y2H) (Bruckner *et al.* 2009; Gnanasekaran and Pappu 2023; Xie *et al.* 2019), co-immunoprecipitation (co-IP) (Evans and Paliashvili 2022; Lin and Lai 2017; Lo Sardo 2023; Masters 2004; Tan and Yammani 2022), and GST-pull down assay (Einarson *et al.* 2007; Kim and Hakoshima 2019). Co-IP is a commonly employed technique for identifying protein interactions and can be applied across various environments, tissues, and cell types. This method enables the identification of direct or indirect interactions within a protein complex. In brief, co-IP involves isolating the bait protein and its complexes from cell lysates using a specific antibody for the bait protein along with beads. A typical co-IP experiment encompasses four key steps: the preparation of cell lysates, the binding of antibodies and bait protein, the purification of protein and antibody complexes, and the removal of non-specifically bound proteins.

Here, we present a co-IP protocol for LD proteins, which combines LD isolation with co-IP procedure to investigate protein interactions on LDs. In this protocol, we introduced two additional steps: the isolation of LDs and the extraction of LD proteins into IP buffer prior to the co-IP procedure. The first step guarantees the co-IP LD protein specifically while the second step removes lipids from co-IP to enhance reliability. This method enables us to directly unveil protein complexes on LDs and gain deeper insights into the functional roles of proteins associated with LDs.

## MATERIALS AND EQUIPMENT

The reagents and equipment used in this protocol are listed in Table 1. The buffers and solutions used in this protocol are listed in Table 2.

**Table 1:** The reagents and equipment used in this protocol

Reagents and equipment	Source	Cat. number
Reagents		
NaCl	Sigma-Aldrich	Cat. #S7653
KCl	Sigma-Aldrich	Cat. #P9333
MgCl_2_	Sigma-Aldrich	Cat. #208337
Sucrose	Sigma-Aldrich	Cat. #S9378
Tricine	Sigma-Aldrich	Cat. #T0377
HEPES	Sigma-Aldrich	Cat. #H3375
Tris	Sigma-Aldrich	Cat. #T1503
Glycerol	Sigma-Aldrich	Cat. #G5516
SDS	Sigma-Aldrich	Cat. #L4390
Bromophenol blue	Sigma-Aldrich	Cat. #B0126
β-ME	Sigma-Aldrich	Cat. #M6250
EDTA	Sigma-Aldrich	Cat. #E1644
Triton X-100	Sigma-Aldrich	Cat. #T8787
Anti-FLAG M2 Affinity beads	Sigma-Aldrich	Cat. #A2220
Protease inhibitor cocktail	Roche	Cat. #5892791001
PMSF	Sigma-Aldrich	Cat. #52332
PBS	Sangon Biotech	Cat. #E607008
OA	Sigma-Aldrich	Cat. #O7501
Equipment		
Ultracentrifuge	Beckman	Optima L-100
Centrifuge	Eppendorf	5424R
Nitrogen bomb	Parr instrument	Cat. #4639
Dounce homogenizer	Wheaton	Cat. #357542

**Table 2:** The buffers and solution used in this protocol

Buffers and solution	Solution formulation
2× Sample Buffer	100 mmol/L Tris-HCl (pH 6.8), 4% (*m*/*v*) SDS, 20% Glycerol (*v*/*v*), 0.04% (*m*/*v*) Bromophenol blue, 4% β-ME
Buffer A	20 mmol/L Tricine, 250 mmol/L Sucrose, pH = 7.8
Buffer B	200 mmol/L HEPES, 100 mmol/L KCl, 2 mmol/L MgCl_2_, pH = 7.4
RIPA 1	50 mmol/L Tris-HCl (pH = 7.5), 150 mmol/L NaCl, 1 mmol/L EDTA, 1% Triton X-100, 10% Glycerol
RIPA2	50 mmol/L Tris-HCl (pH = 7.5), 300 mmol/L NaCl, 1 mmol/L EDTA, 1% Triton X-100, 10% Glycerol
RIPA3	50 mmol/L Tris-HCl (pH = 7.5), 500 mmol/L NaCl, 1 mmol/L EDTA, 1% Triton X-100, 10% Glycerol

## PROCEDURE

**Figure 1:**
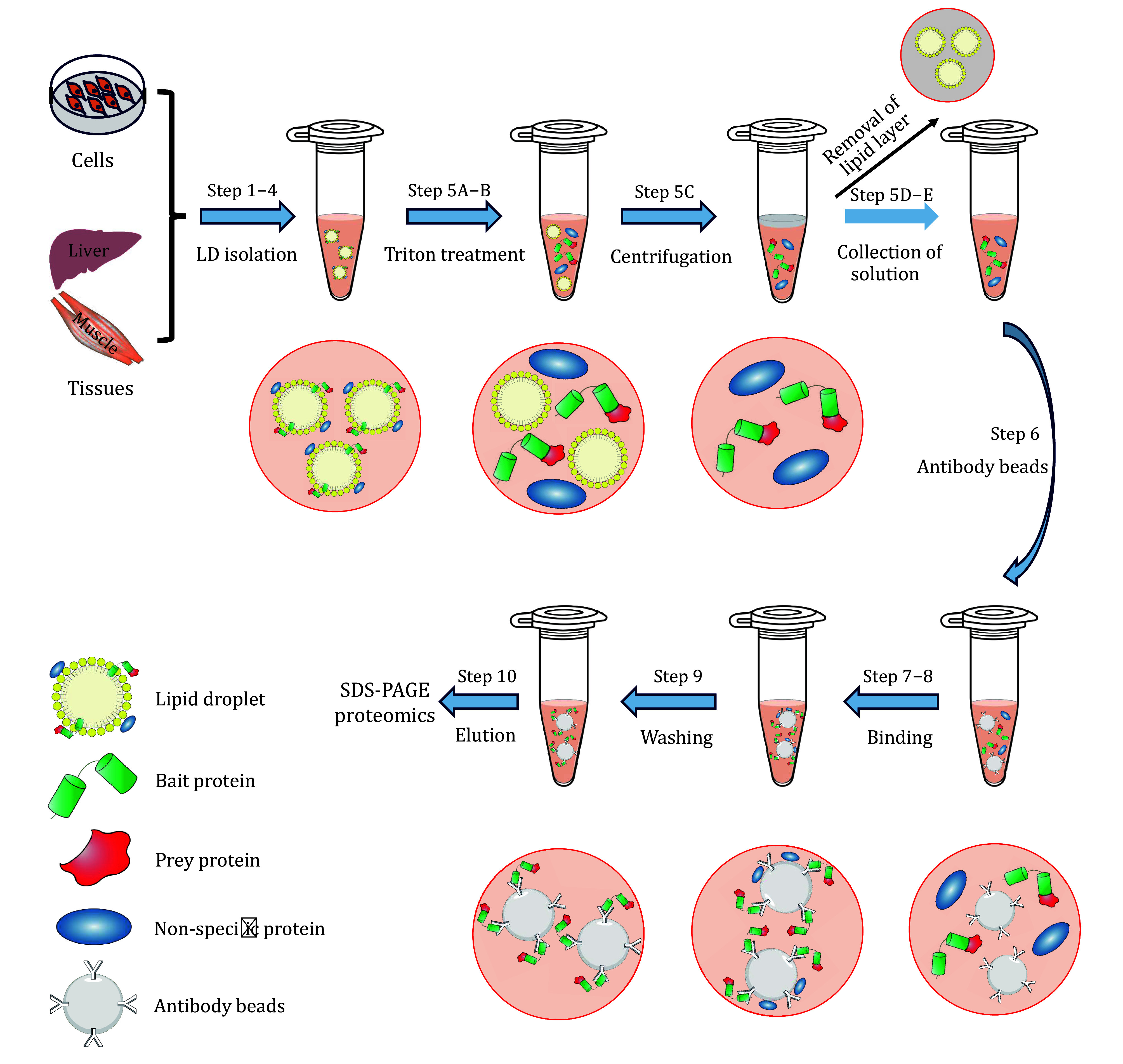
The procedure of lipid droplet protein co-immunoprecipitation. The experimental process includes ten steps that are indicated in the flowchart. These steps can be summarized into seven key components as illustrated in the figure. They are LD isolation, treatment LD with Triton, centrifugal separation of the lipid layer and protein solution, removal of the lipid layer and collection of LD protein-containing solution, binding of LD proteins to antibody beads, washing of non-specific proteins, and elution of the bait-prey complex. The co-IP samples obtained from the experiment are used for SDS-PAGE analysis and MS-based proteomics

The procedure of this protocol is shown in Fig. 1 in the form of a flow chart.

### Lipid droplet isolation

LDs are isolated using an adapted approach based on the method we originally developed (Ding *et al.* 2013).

#### Step 1: Cell collection

(A) When the cells reach 90% confluence in a 100-mm cell culture plate, introduce oleic acid (OA) to the cell culture and incubate for 12 hours to facilitate the accumulation of LDs.

**[Note]** The specific number of dishes may vary depending on the cell type and its lipid droplet content. In this protocol, we illustrate the procedure using a FLAG-Rab18 overexpressing C2C12 cell line as an example. In this protocol, it is required to use approximately 10-mm cell culture dishes for each experimental group.

(B) Rinse the cells with ice-cold PBS and collect the cells using a cell scraper. Transfer the collected cells into a centrifuge tube and centrifuge them at 500 *g* for 10 min at 4 °C.

#### Step 2: Cell disruption

(A) Suspend the cells in Buffer A supplemented with 0.1 mmol/L PMSF (phenylmethylsulfonyl fluoride) and maintain the suspension on ice for 20 min.

(B) Utilize a nitrogen bomb to disrupt the cells under a pressure of 750 psi for 20 min, while keeping the process on ice.

(C) Centrifuge the cell lysates at 1000 *g* for 10 min at 4 °C to remove nuclei, cell debris and unbroken cells. The resulting supernatant is the postnuclear supernatant (PNS) fraction.

#### Step 3: LD collection

(A) Transfer 10 mL of the PNS fraction into a SW41 tube and then carefully layer 1.5 mL of Buffer B on top of the PNS.

(B) Centrifuge the PNS at 250,000 *g* for 1 h at 4 °C. After centrifugation, LDs will float on the top layer of the liquid.

(C) Carefully collect LDs from the top layer and transfer them into a 1.5-mL centrifuge tube.

#### Step 4: Washing the LD fraction

(A) Centrifuge the LDs at 20,000 *g* for 5 min at 4 °C.

(B) Carefully remove and discard the underlying solution and pellet, using a 200 µL long tip to avoid disturbing the LDs.

(C) Add 200 µL of Buffer B to resuspend the LDs, then centrifuge them at 20,000 *g* for 5 min at 4 °C. Remove the underlying solution and pellet.

(D) Repeat Step 4C two more times to ensure thorough washing of LDs.

**[Note]** Obtaining high-quality LDs is a crucial prerequisite for the success of a downstream LD protein co-IP experiment. For detailed instructions on how to purify LDs with high quality from various species, please refer to our previous protocol (Ding *et al.* 2013).

### Co-immunoprecipitation of LD-associated proteins

#### Step 5: Extraction of lipid droplet proteins

(A) Carefully remove Buffer B from the purified LD fraction. Add 0.5 mL of ice-cold RIPA1 into the LDs and gently resuspend them. Prior to use, ensure that RIPA1 is supplemented with 1× protease inhibitor cocktail and 0.1 mmol/L PMSF.

(B) Place the solution onto a rotating wheel set to gentle rotation at 4 °C for 30 min. The recommended rotation speed is 10 r/min, as higher speeds may disrupt protein interactions/complexes.

(C) After rotation, centrifuge the LD solution at 20,000 *g* for 10 min at 4 °C.

(D) Carefully remove the top layer, which contains most of the lipids, and transfer the remaining LD protein solution into a fresh 1.5 mL centrifuge tube.

(E) Take 50 µL of the LD protein solution to be used as a co-IP input sample and add 50 µL 2× Sample Buffer to it for further analysis.

#### Step 6: Blocking anti-FLAG M2 agarose beads

(A) For each sample, take 30 µL of anti-FLAG M2 beads and add 1 mL of RIPA1 containing 200 µg/mL of BSA to block the beads.

**[Note]** In this protocol, commercial antibody-conjugated beads are utilized. However, it's worth noting that you can also prepare the beads by binding the specific antibody to beads that have been pre-coated with Protein A/G or anti-IgG antibodies.

(B) Place the beads on a rotating wheel set to gentle rotation at 4 °C for 10 min at a speed of 10 r/min.

(C) Centrifuge the beads at 1000 *g* for 1 min at 4 °C.

(D) Carefully remove the solution from the beads and add 1 mL of RIPA1 containing 200 µg/mL of BSA to block the beads again. Repeat this blocking step a total of three times to ensure thorough blocking of the beads.

#### Step 7: Incubation of LD proteins with antibody-conjugated beads

(A) Following the final blocking step, carefully remove the blocking solution. Then, add the LD protein solution obtained from Step 5D into the beads.

(B) Incubate the sample mix at 4 °C for 3 h with gentle rotation. During this incubation, the bait protein will bind to the antibodies on the beads.

#### Step 8: Collection of beads binding the antibody-LD protein complexes

(A) After incubation, centrifuge the sample at 1000 *g* for 1 min at 4 °C.

(B) Transfer all of the supernatant into a new 1.5-mL centrifuge tube. Take 50 µL of the supernatant to be used as the co-IP supernatant sample and add 50 µL of 2× Sample Buffer to it for further analysis.

#### Step 9: Washing away non-specifically binding proteins

(A) Add 1 mL of RIPA3 containing 1× protease inhibitor cocktail and 0.1 mmol/L PMSF into the beads. Wash the beads at 4 °C for 10 min with gentle rotation. Centrifuge the beads at 1000 *g* for 1 min at 4 °C, and then carefully remove the supernatant.

(B) Repeat Step 9A once.

(C) Add 1 mL of RIPA2 with 1× protease inhibitor cocktail and 0.1 mmol/L PMSF into the beads. Wash the beads at 4 °C for 10 min with gentle rotation. Centrifuge the beads at 1000 *g* for 1 min at 4 °C, and then carefully remove the supernatant.

(D) Repeat Step 9C once.

(E) Add 1 mL of RIPA1 with 1× protease inhibitor cocktail and 0.1 mmol/L PMSF into the beads. Wash the beads at 4 °C for 10 min with gentle rotation. Centrifuge the beads at 1000 *g* for 1 min at 4 °C, and then carefully remove the supernatant.

(F) Repeat Step 9E once.

#### Step 10: Separation and silver staining of the LD protein complexes from the beads

(A) Add 50 µL of 2× Sample Buffer into the beads as the co-IP IP sample.

(B) Heat the input, supernatant, and IP samples at 95 °C for 5 min to denature. Then perform SDS-PAGE.

(C) Following SDS-PAGE, transfer the gel to a 15-cm dish containing fixative solution and let the gel fix for 30 min.

(D) Discard the fixative solution and immerse the gel in a sensitizing solution for 30 min.

(E) Remove the sensitizing solution and wash the gel using ddH_2_O four times, with each wash lasting 5 min.

(F) Apply silver staining solution to the gel and allow it to stain for 20 min.

(G) Add a chromogenic solution until clear bands become visible on the gel.

(H) Conclude the staining process by adding a stopping solution. Then selectively excise the bands that are unique to the LD co-IP experimental group in comparison to the LD control group, indicating proteins binding to the bait protein. These excised bands can be further prepared for MS analysis to identify the proteins associated specifically with the bait protein.

**[Note]** The method to separate the bound proteins from the beads depends on the specific detection method you intend to use for subsequent protein analysis. There are various approaches for analyzing the isolated protein of interest and its interacting partners on LDs, including silver staining after SDS-PAGE, MS, enzymatic assays, and more. The choice of buffer or treatment should align with your experimental objectives. For instance, when conducting MS analysis on the proteins bound to the beads, you can directly introduce 50 µL of 8 mol/L urea to the beads.

## ANTICIPATED RESULTS

**Figure 2:**
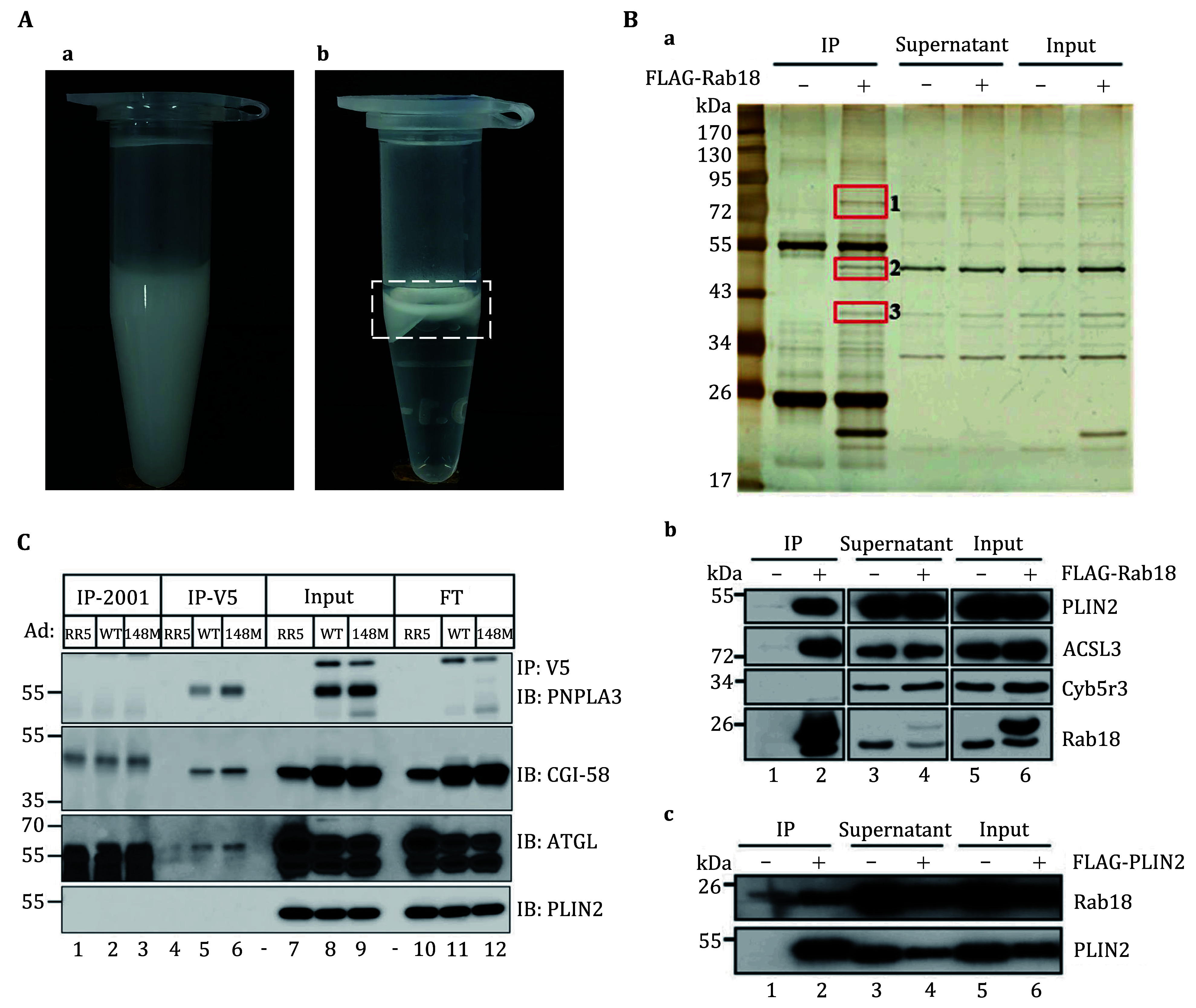
Anticipated results of lipid droplet co-immunoprecipitation assay. **A** The isolated LDs are collected in a 1.5 mL centrifuge tube. High-quality isolated LDs should present as a milky homogenate (a). The isolated LDs after RIPA1 treatment and centrifugation. The LD proteins are extracted into the solution, and after centrifugation, the LD lipids typically form a distinct top layer in the solution (b), as indicated in the white box. **B** LDs were isolated from OA-treated WT and FLAG-Rab18 overexpressing C2C12 cells. Immunoprecipitation was performed on the LD proteins with anti-FLAG M2 beads followed by analysis with silver staining (a). Western blot analysis of the precipitates pulled down by FLAG-Rab18 (b). Western blot analysis of the precipitates pulled down by FLAG-PLIN2 (c). The figure is reprinted from Deng *et al.* (2021). **C** Co-immunoprecipitation of PNPLA3 (WT and 148M) and CGI-58. The hepatic LDs were isolated from female mice that were infected with Ad-RR5 or Ad PNPLA3-V5 (WT or 148M). The figure is reprinted from Wang *et al.* (2019)

In this approach, we perform co-IP experiments directly using proteins from isolated LDs instead of whole cells to identify protein–protein interactions or protein complexes associated with LDs. This method minimizes the influence of other cellular components and aids in the identification of specific protein–protein interactions or protein complexes associated with LDs. While the purification of LDs may lead to the loss of some proteins with weaker interactions or less stability on LDs, this method significantly reduces interference from other cellular constituents. This, in turn, facilitates the identification of specific protein–protein interactions or protein complexes that are uniquely associated with LDs. Therefore, ensuring the purification of high-quality LDs is essential for the success of this method. LDs, due to their lower density, should rise to the top of cell lysates during centrifugation, as depicted in Fig. 2A. The isolated LDs should exhibit a well-suspended, milky emulsion appearance, while contamination from other cellular membranes must be minimized. Detailed instructions for obtaining high-quality LDs can be found in our previous protocol (Ding *et al.* 2013). Subsequently, during the co-IP procedure conducted on isolated LDs, LD proteins are extracted into the solution following treatment with detergent and centrifugation. Meanwhile, the neutral lipids from LDs remain in the upper layer of the solution, as illustrated in Fig. 2A. To enhance the resulting specificity, it is crucial to discard the neutral lipids to reduce non-specific protein binding.

In this protocol, we use a FLAG-Rab18 overexpressing cell line as an example, providing a comprehensive procedure that combines LD isolation and co-IP experiments to identify protein–protein interactions on LDs. As depicted in Fig. 2B, three unique bands bind to Rab18 on LDs compared to the control. These bands were analyzed by mass spectrometry and the results reveal that the proteins associating with Rab18 in the LD co-IP experiment predominantly consist of LD-associated proteins (Deng *et al.* 2021). These proteins represent potential candidates for interacting with Rab18 on LDs. In fact, through the application of this methodology, we have successfully identified a complex involving ADRP, ACSL3, and Rab18 on C2C12 LDs, as shown in Fig. 2B (Deng *et al.* 2021). This method has also been employed to unveil the interaction between PNPLA3 and CGI-58 on LDs (Fig. 2C) (Wang *et al.* 2019). We believe that this protocol will significantly facilitate the discovery and investigation of proteins associated with lipid droplets.

## Conflict of interest

Xiaochuan Fu, Shuyan Zhang and Pingsheng Liu declare that they have no conflict of interest.
